# Supplementary material for: The interactome of intact mitochondria by cross-linking mass spectrometry provides evidence for coexisting respiratory supercomplexes
Source: Mol Cell Proteomics. 2017 Dec 8;17(2):216–32. doi: 10.1074/mcp.RA117.000470 (PMC5795388; doi:10.1074/mcp.RA117.000470)
Supplement: Supplemental Data [file supp_RA117.000470_133922_0_supp_23978_fzffwf.zip › spectra_annotation/mito_DR_spectra_annotation/70-1-7-1-13-1.pdf]

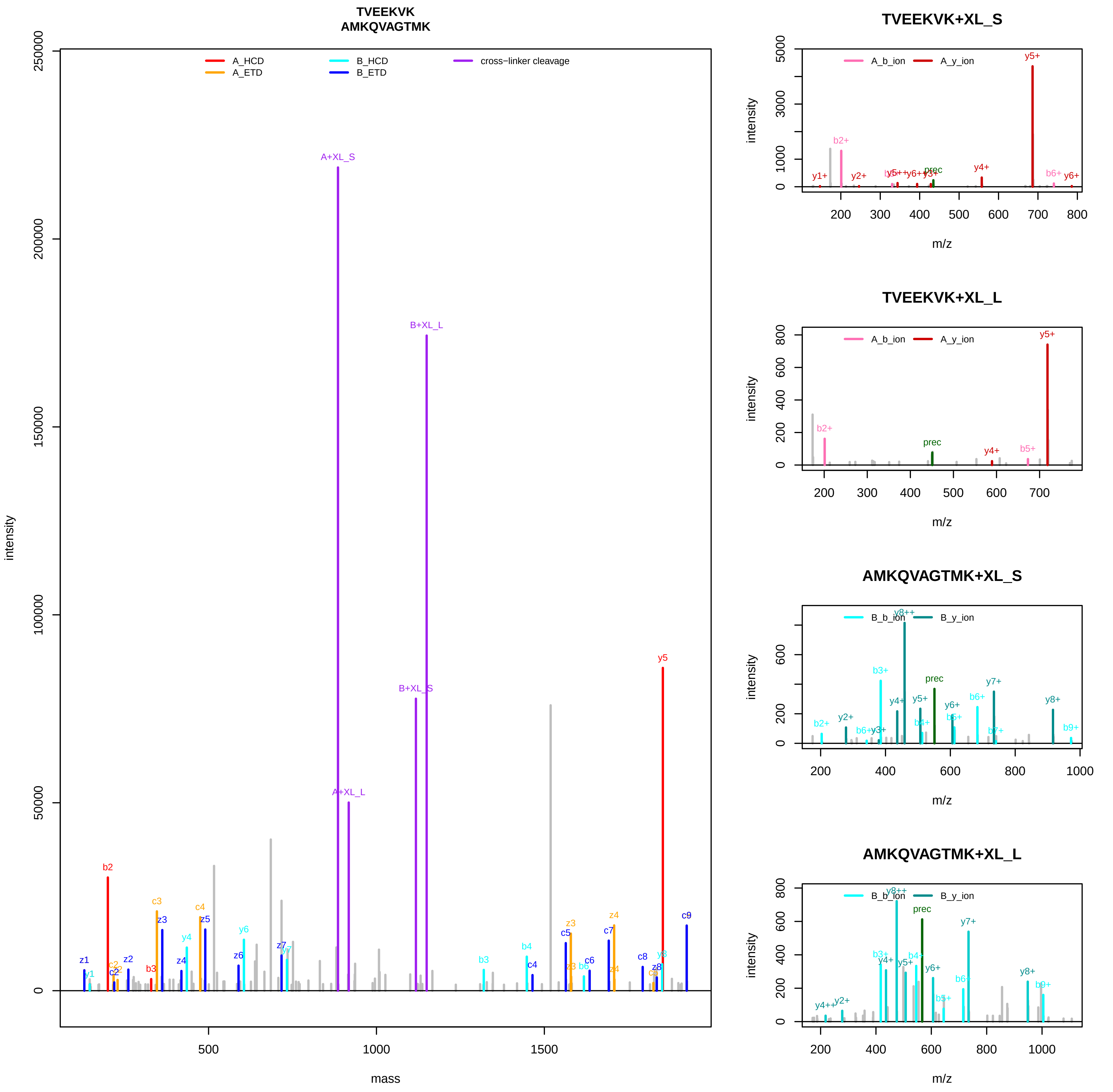
TVEEKVK
AMKQVAGTMK
A_HCD
A_ETD
B_HCD
B_ETD
cross−linker cleavage
A+XL_S
B+XL_L
B+XL_S
A+XL_L
y5
b2
c3
c4
z5
z3
y4
y6
z1
z2
z4
z6
z7
b3
c2
b4
c4
c5
c6
c7
c8
z8
c9
intensity
mass
0
50000
100000
150000
200000
250000
500
1000
1500
TVEEKVK+XL_S
A_b_ion
A_y_ion
y5+
b2+
y1+
y2+
y4+
prec
b6+
y6+
intensity
m/z
TVEEKVK+XL_L
b2+
prec
y4+
b5+
AMKQVAGTMK+XL_S
B_b_ion
B_y_ion
y8++
b3+
prec
y7+
y4+
y5+
y6+
b6+
y8+
b2+
y2+
b9+
AMKQVAGTMK+XL_L
y7+
y6+
b6+
y8+
b9+
y2+
